# Comparative stability of Major Facilitator Superfamily transport proteins

**DOI:** 10.1007/s00249-017-1197-7

**Published:** 2017-01-23

**Authors:** Nicola J. Harris, Heather E. Findlay, Michael R. Sanders, Mateusz Kedzierski, Ália dos Santos, Paula J. Booth

**Affiliations:** Department of Chemistry, King’s College London, Britannia House, 7 Trinity Street, London, UK

**Keywords:** Circular dichroism, Major Facilitator Superfamily transporters

## Abstract

Membrane transporters are a vital class of proteins for which there is little available structural and thermodynamic information. The Major Facilitator Superfamily (MFS) is a large group of transport proteins responsible for transporting a wide range of substrates in eukaryotes and prokaryotes. We have used far-UV circular dichroism (CD) to assess whether transporters from this superfamily have the same chemical and thermal stability. We have compared the stability of five different MFS transporters; PepT_So_ from *Shewanella oneidensis* and LacY, GalP, GlpT and XylE from *Escherichia coli*, as well as a known stable mutant of LacY, LacY-C154G. CD stability measurements revealed that these transporters fall into two broad categories. The ‘urea-sensitive’ category includes LacY-WT, GalP and GlpT, which each lose around a third of their secondary structure in 8 M urea and two-thirds in the harsher denaturant guanidine hydrochloride (GuHCl). The ‘urea-resistant’ category includes LacY-C154G, XylE and PepT_So_. These resistant transporters lose very little secondary structure in 8 M urea, and LacY-C154G and PepT_So_ resist denaturation by GuHCl up to a concentration of 4 M. The stabilities of LacY, GlpT, XylE and PepT_So_ correlated with their crystal structure conformations, implying that a similar conformation is adopted in vitro. The ‘urea-sensitive’ transporters LacY and GlpT were crystallised inward-open states, while XylE and PepT_So_ were crystallised in occluded states. This study highlights the importance of studying a wide range of similar proteins, as a similar secondary structure and overall function does not necessarily confer the same stability in vitro.

**Electronic supplementary material:**

The online version of this article (doi:10.1007/s00249-017-1197-7) contains supplementary material, which is available to authorized users.

## Introduction

**Fig. 1:**
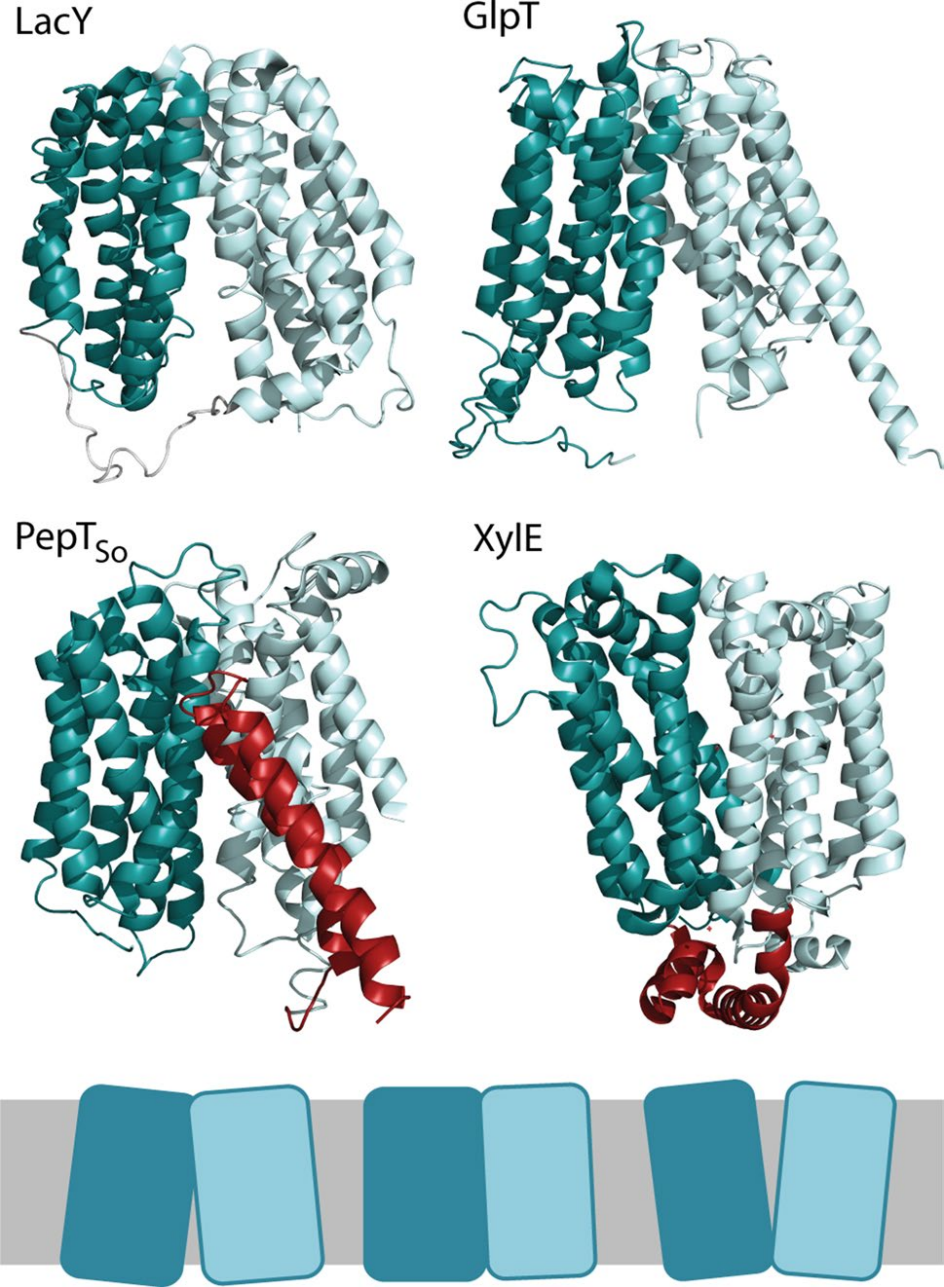
MFS transporters share a common fold. MFS transporters are comprised of two six helix bundles arranged in two domains. LacY (*top left*, PDB 2V8 N) and GlpT (*top right*, PDB 1PW4) have been solved in an inward open state and PepT_So_ (*bottom left*, PDB 2XUT) in an occluded state. XylE (*bottom right*, PDB 4GBY) has been solved in a range of partially occluded states, the one shown here is partially occluded-outward facing. Both PepT_So_ and XylE have extra helices (shown in *red*) within the connecting loop between the N and C domains; in PepT_So_ the helices are HA and HB, in XylE they are known as the ICH domain. All of these MFS structures have the N domain on the *left*, the C domain on the *right* and are oriented with the cytoplasmic side at the *bottom* of the structure. Below is a *cartoon* of the MFS transporter structure, showing different conformations: inward facing, occluded and outward facing

The Major Facilitator Superfamily is a large group of secondary transporters found in all organisms, responsible for transporting a wide range of substrates (Yan 2015). Whether uniporter, symporter or antiporter, they share a common fold; two alpha helical domains of six helices each with ‘3 + 3’ symmetry, connected by a cytoplasmic loop (Fig. 1). Some MFS subfamilies have additional helices which are not a part of the N and C domains; the sugar porter (SP) family have the ICH domain (Deng et al. 2014; 2015; Nomura et al. 2015; Wisedchaisri et al. 2014), and some proton:oligopeptide transporters (POT) have two extra helices designated HA and HB (Doki et al. 2013; Newstead et al. 2011; Solcan et al. 2012; Zhao et al. 2014). To date, there now are more than twenty unique MFS transporters with a high resolution crystal structure, including examples from both prokaryotes and eukaryotes.

The current model for MFS substrate transport is the alternating access mechanism. Although the majority of the MFS high resolution structures are in an inward-facing conformation (Yan 2015), there are now a number of structures representing the different conformational states of the transport cycle (Deng et al. 2014, 2015; Nomura et al. 2015). Briefly, the substrate accesses the substrate binding site when the MFS transporter is in an outward-open conformation. Substrate binding then induces a conformational change in the transporter to an inward-open state, via an occluded ligand-bound state. The substrate is then released inside the cell, and the transporter returns to an outward-facing conformation via an occluded, non-bound state.

Membrane proteins, and transporters in particular, are very difficult to study in vitro due to their hydrophobic nature rendering them highly unstable once removed from their native membrane environment. In the case of transporters, the flexibility that is essential for their function further increases their instability in vitro. There is therefore very little information available on the stability of transporters. High resolution structures of mammalian MFS transporters have only recently been solved, after considerable effort (Deng et al. 2014, 2015; Nomura et al. 2015). There are just two examples of high resolution structures of human transporters, GLUT1 and GLUT3, highlighting the need for further study on the stability of transporters in vitro.

Far-UV circular dichroism (CD) is a commonly used spectroscopic technique, which can provide information on the secondary structure content of proteins in the 170–240 nm wavelength range. The use of Synchrotron Radiation CD (SRCD) is particularly useful, as the high light flux of the synchrotron source enables measurement to lower wavelengths than an in-house machine and gives better quality data with a better signal to noise ratio. SRCD therefore enables measurements of membrane proteins at the lower protein concentrations at which they can typically be worked with, and also minimises the artefacts which arise from absorbance of the sample. The more comprehensive wavelength range also allows for more accurate deconvolution of secondary structure. There have been many recent advances in the use of CD on membrane proteins, including the development of a reference dataset, SMP180, which contains membrane protein structural information to aid deconvolution of CD spectra for more accurate structure determination (Abdul-Gader et al. 2011). The use of oriented CD (OCD) is being used increasingly to ascertain the orientation of α-helices with respect to the membrane (Burck et al. 2016; Ulmschneider et al. 2014), distinguishing between helices inserted in the bilayer and helices oriented in the plane of the membrane.

CD spectra of proteins which are predominantly α-helical, such as MFS transporters, have a characteristic shape; a positive band around 190 nm, and two negative bands at 208 and 222 nm. By monitoring the change in the 222 nm band, the intrinsic stability of a protein upon addition of a denaturant can be assessed. By using CD to compare the stability of different transporters of the MFS, we can directly compare the secondary structure changes induced upon denaturation without the difficulties in interpretation which can arise from other techniques such as fluorescence. A clear advantage of CD for membrane protein stability and folding studies is that it definitively measures changes in helical structure, thus proving loss and regain of structure. In contrast, changes in intrinsic protein fluorescence do not necessarily arise from protein structural changes, and can be due to alterations in the local solvent environment of aromatic amino acids.

We have used CD measurements of chemical denaturation to assess whether the similar structure of the MFS transporters confers similar intrinsic properties. Previous work on the *Escherichia coli* lactose transporter LacY (Harris et al. 2014) and the *E. coli* galactose transporter GalP (Findlay et al. 2010) has shown that when in detergent micelles, LacY and GalP both unfold reversibly in the chaotrope urea, with a very similar free energy of unfolding observed (Δ*G*_unfolding_ = ~2 kcal mol^−1^). In this paper, we have expanded these stability studies by comparing LacY and GalP with the *E. coli* glycerol-3-phosphate transporter GlpT and the *E. coli* xylose transporter XylE, a homologue to the GLUT transporters (Sun et al. 2012). We have included the dipeptide transporter PepT_So_ from *Shewanella oneidensis*, a homologue to the human dipeptide transporters PepT1 and PepT2 (Newstead et al. 2011). We have also measured the stability of a stable mutant of LacY, C154G, a mutant found to have higher thermal stability and decreased conformational flexibility than the wild-type (WT) protein (Smirnova and Kaback 2003).

Interestingly, despite the similar structure of the MFS transporters studied, there are significant differences in stability to chemical denaturant between the different transporters, indicating that behaviour in vitro cannot be generalised across a whole family of proteins. This highlights the need to study as many membrane proteins as possible, in order to find the factors that govern stability in vitro and facilitate the study of transporters with a role in health and disease.

## Methods

### Materials

All the standard reagents used were purchased from Sigma–Aldrich or Fisher Scientific UK Ltd. All reagents used were of the highest available grade. The >99.5% *n*-Dodecyl-*b*-d-Maltopyranoside (DDM) was purchased from Generon. PepT_So_ was cloned from pWaldo into pET-28a modified with a 10-His tag for protein overexpression. The pWaldo-GFPe_PepTSo was a gift from So Iwata and Simon Newstead (Addgene plasmid # 58334).

### Overexpression and purification of MFS transporters

All of the MFS transporters were overexpressed in *E. coli* BL21-AI, using the vector pET28a modified with a 10-His tag. The purification procedures for LacY-WT and GalP have been published previously (Findlay et al. 2010; Harris et al. 2014). The LacY-WT purification procedure was followed for the overexpression and purification of LacY-C154G, GlpT, PepT_So_ and XylE, but with the addition of 150 mM NaCl to all buffers for PepT_So_ purification. GlpT was purified with 50 mM TrisHCl pH 7.4 throughout instead of sodium phosphate buffers. Typically, 2 mg of purified PepT_So_ per 1 L of LB was produced, and around 1.5 mg of GlpT, LacY and XylE per 1 L of LB.

### Circular dichroism measurements

CD chemical denaturation was performed and analysed following the method described (Findlay et al. 2010; Harris et al. 2014), using CDtool (Lees et al. 2004) and the Dichroweb server (Whitmore and Wallace 2004) to analyse the spectra. A 5 min incubation in denaturant was sufficient to reach equilibrium for all the transporters, apart from PepT_So_ which required 20 min in guanidine hydrochloride (GuHCl) to unfold. Each MFS transporter was measured at a protein concentration from 0.1 to 3.5 mg.ml^−1^, in 0.01–0.2 mm pathlength cells. Synchrotron Radiation CD (SRCD) was measured using the CD12 beamline at ANKA (Karlsruhe Institute of Technology, Germany).

CD measurements of GlpT with 50 µM of the ligand pyridoxal-5-phosphate (P5P) were measured at a protein concentration of 0.01 mg ml^−1^ and a cell pathlength of 2 mm. XylE CD measurements with 1 mM xylose were measured at a protein concentration of 0.35 mg ml^−1^, and a cell pathlength of 0.2 mm. LacY measurements with 10 mM lactose was measured at a protein concentration of 0.3 mg ml^−1^ and a cell pathlength of 0.2 mm.

## Results

### Secondary structure loss in urea

All MFS transporters have a very similar secondary structure; two bundles of six α-helices connected by a cytoplasmic loop, with the substrate binding site in between the two domains. The two domains are commonly referred to as the ‘N’ and ‘C’ domains. The CD spectra of all the MFS transporters studied are therefore very similar, and are 70–90% α-helical when the secondary structure content is calculated using the Dichroweb server and the SMP180 reference dataset (Abdul-Gader et al. 2011; Whitmore and Wallace 2004) (Fig. 1, Fig. S1).

**Fig. 2:**
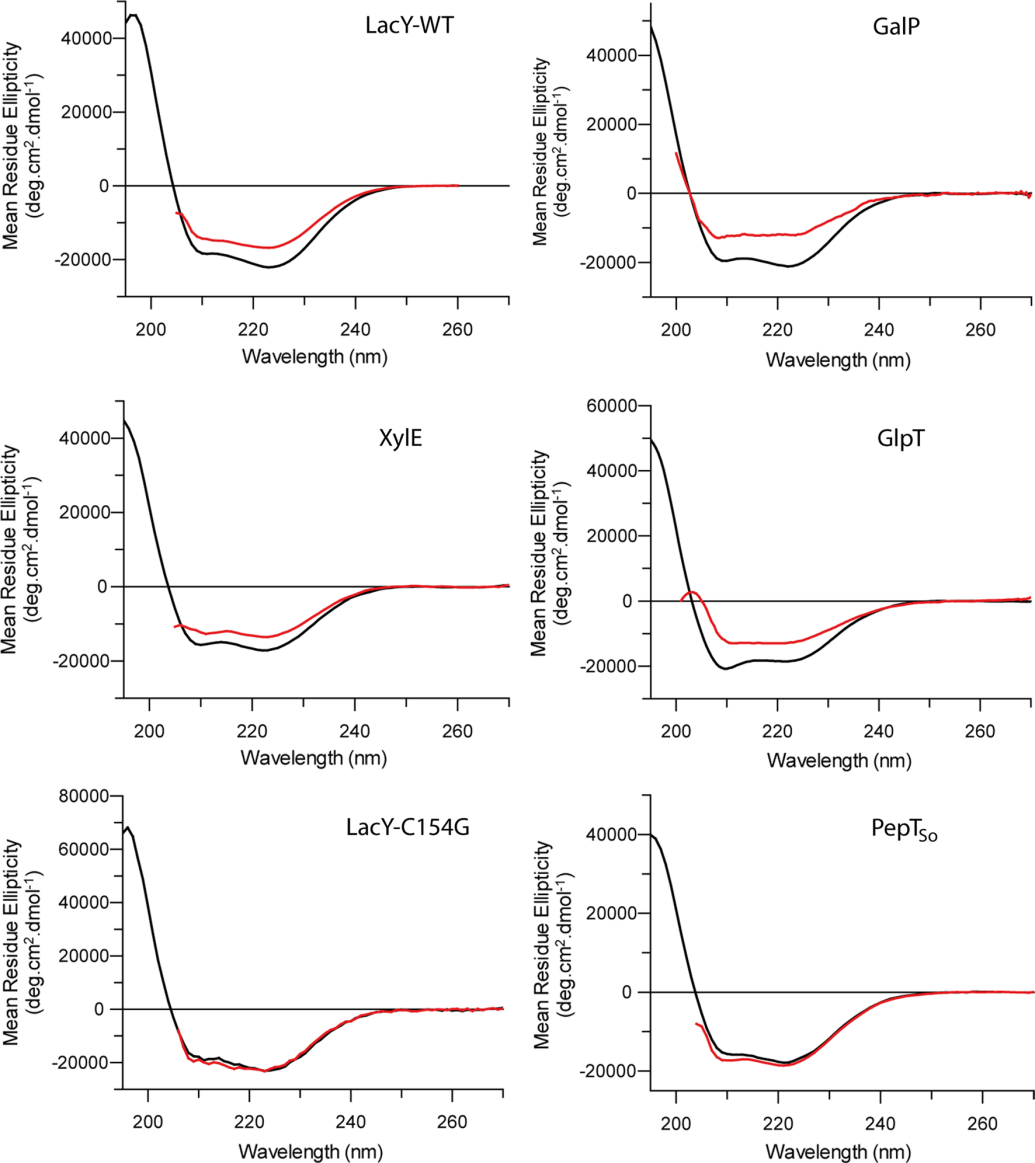
MFS transporter unfolding in urea. LacY-WT, GalP, XylE, GlpT, LacY-C154G and PepT_So_ were incubated in either buffer (*black lines*) or 8 M urea (*red lines*), and the secondary structure measured by CD. LacY-C154G and PepT_So_ do not unfold in 8 M urea, in contrast to LacY-WT, GalP and GlpT which all lose around a third of their secondary structure in 8 M urea. XylE retains around 85% of its helical structure in 8 M urea. The LacY-WT and GalP data have both been published previously (Findlay et al. 2010; Harris et al. 2014)

**Fig. 3:**
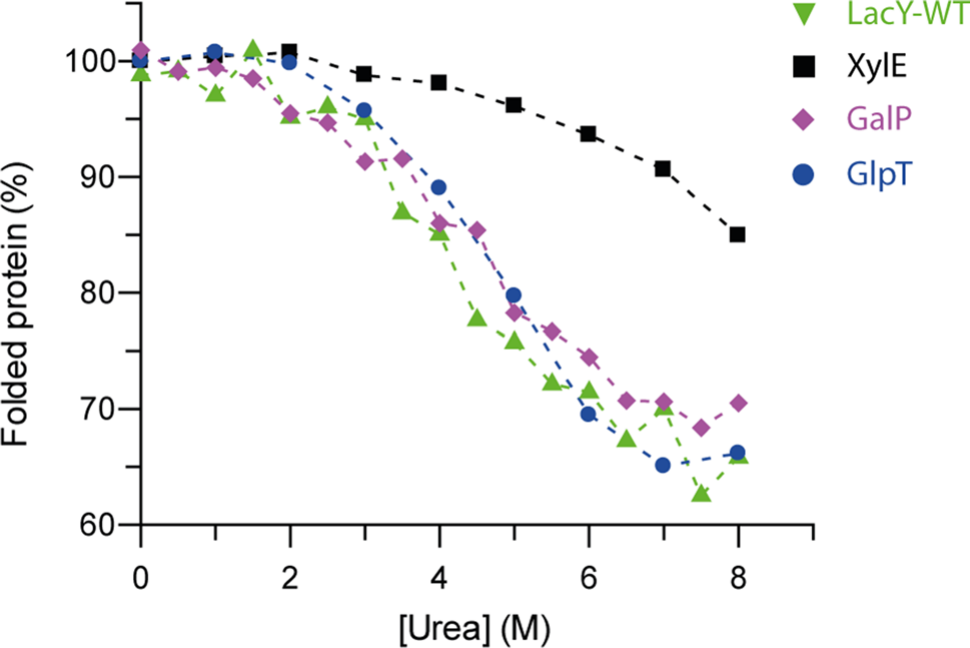
XylE unfolds at higher urea concentrations than the transporters LacY-WT, GalP and GlpT. The amount of secondary structure of LacY-WT, GalP, GlpT and XylE was measured by CD at increasing urea concentration. The unfolding of LacY-WT [*green triangles*, previous data (Harris et al. 2014)], GalP [(*pink diamonds*, previous data, (Findlay et al. 2010)] and GlpT (*blue circles*) each begin to unfold in 2.5 M urea, and the unfolding transition is complete in 8 M urea. In contrast XylE (*black squares*) unfolds much less, with significant unfolding occurring only above 5 M urea. Each is shown as  % folded protein, with 100% being the CD signal intensity at 222 nm in the native fold

Previous work has demonstrated that LacY-WT and GalP both unfold reversibly in urea (Findlay et al. 2010; Harris et al. 2014). The unfolding transition for each begins in 2 M urea, with a midpoint of around 4 M, and both LacY-WT and GalP retain around 60% of their helical structure in 8 M urea (Figs. 2, 3; Table 1). To ascertain whether other MFS transporters exhibit the same behaviour, the transporters GlpT, PepT_So_, LacY-C154G and XylE were each unfolded in urea, and measured by CD to assess the loss of secondary structure (Figs. 2, 3). GlpT required slightly higher urea concentrations for unfolding than GalP and LacY, beginning to lose helical structure when the urea concentration was above 4 M, but still losing around a third of its alpha helical content in 8 M urea. PepT_So_ and LacY-C154G however did not lose any secondary structure in urea, even up to a concentration of 8 M urea (Fig. 2). The MFS transporters therefore fall into two broad categories: ‘urea-sensitive’, and ‘urea-resistant’. The urea-sensitive transporters all reach an unfolding transition endpoint in 8 M urea, with the amount of structure lost plateauing at 6 M urea (Fig. 3). XylE was found to be largely urea-resistant, unfolding only when the urea concentration was above 5 M, and retaining around 85% of its secondary structure in 8 M urea (Fig. 3). An accurate estimation of the amount of helical structure remaining in 8 M urea is not possible, due to the high absorbance of urea below 210 nm preventing accurate measurement of the CD signal. It is worth noting that none of these transporters unfold in SDS, regardless of urea stability, as observed by both SDS-PAGE and CD (Fig. S2; Findlay et al. 2010).

**Table 1:** Summary of MFS transporter stability

		Crystal structure conformation	Concentration of urea required for unfolding (M)	Concentration of GuHCl required for unfolding (M)	Effect of ligand on stability
Urea-sensitive	LacY-WT	Inward-open	2 M	1.5 M	No effect
	GalP	Not yet solved	2 M	1.5 M	*
	GlpT	Inward-open	4 M	1.5 M	Unfolds in urea concentrations >6 M when 50 µM P5P present
Urea-resistant	XylE	Solved in various conformations: Partially occluded/inward-open Partially occluded/outward-open Inward-open	5 M	1.5 M	Behaves much more like LacY-C154G and PepT_So_ when 1 mM xylose present; XylE no longer unfolds in urea, and unfolds in GuHCl concentrations >5 M
	PepT_So_	Occluded	No unfolding observed	4 M	–
	LacY-C154G	Inward-open	No unfolding observed	4 M	–

* No data on the effect of ligand on GalP as all the data in this paper are reproduced from (Findlay et al. 2010)

#### Unfolding in the harsher denaturant guanidine hydrochloride

**Fig. 4:**
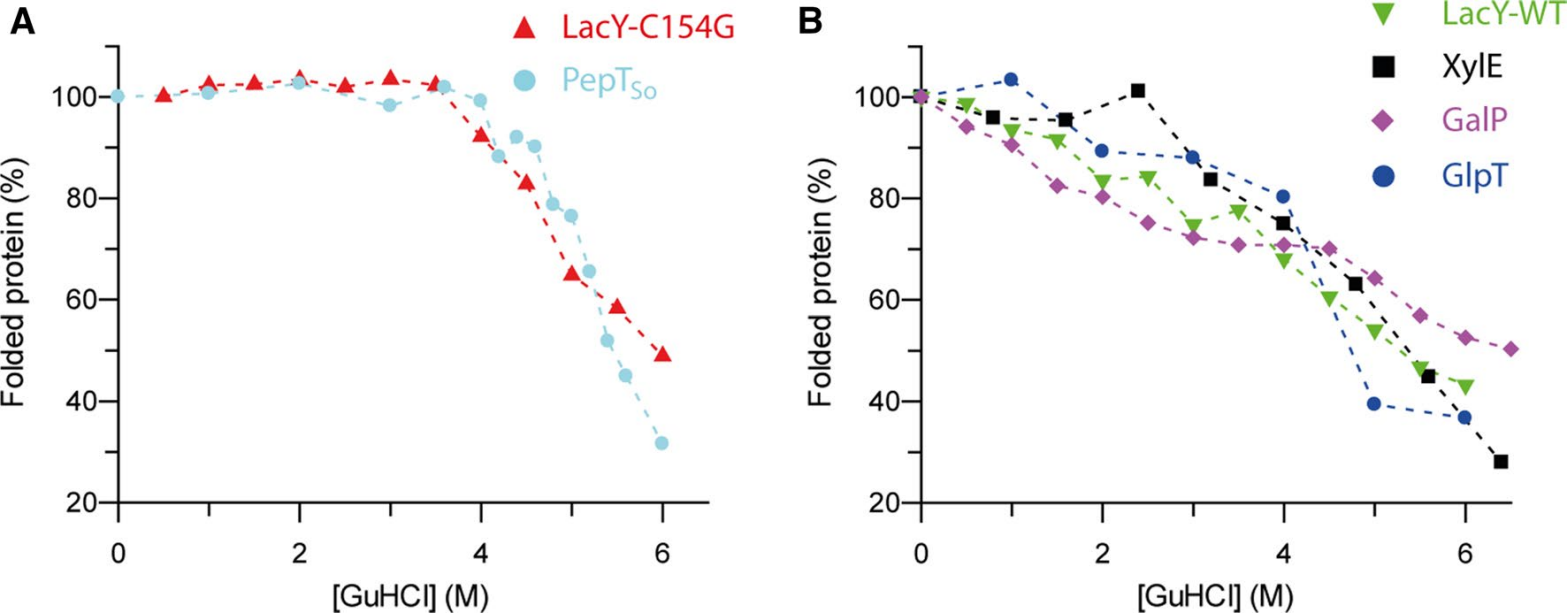
MFS transporters have different stability in GuHCl. **a** The urea-resistant transporters PepT_So_ (*light blue circles*) and LacY-C154G (*red triangles*) have an almost identical dependence on GuHCl. Each is resistant to GuHCl denaturation up to 4 M, at which point there is a steep unfolding transition. **b** In contrast to PepT_So_ and LacY-C154G, LacY-WT (*green triangles*), GalP (*pink diamonds*), XylE (*black squares*) and GlpT (*blue circles*) all unfold steadily as the GuHCl concentration is increased. Regardless of stability, each transporter has a similar amount of secondary structure remaining in 6 M GuHCl, around 30–50%. The GalP data has been published previously in (Findlay et al. 2010)

To further assess and compare MFS transporter stability, each transporter was unfolded in the harsher denaturant guanidine hydrochloride (GuHCl; Fig. 4; Table 1). As expected, the urea-sensitive MFS transporters LacY-WT, GalP and GlpT each unfold rapidly in GuHCl, retaining only 30% of their helical structure in 6 M GuHCl. XylE, which was largely stable to urea, also unfolded in this manner. The urea-resistant PepT_So_ and LacY-C154G are resistant to GuHCl denaturation up to a concentration of 4 M GuHCl. Above this GuHCl concentration, they both rapidly unfold until only around a third of their secondary structure remains in 6 M GuHCl, the same as the urea-sensitive transporters. PepT_So_ differed from the other transporters studied here as it required a 20 min incubation in GuHCl for the denaturation reaction to reach equilibrium, in contrast to a 5 min incubation for the other MFS transporters.

### Addition of ligand increases transporter stability

**Fig. 5:**
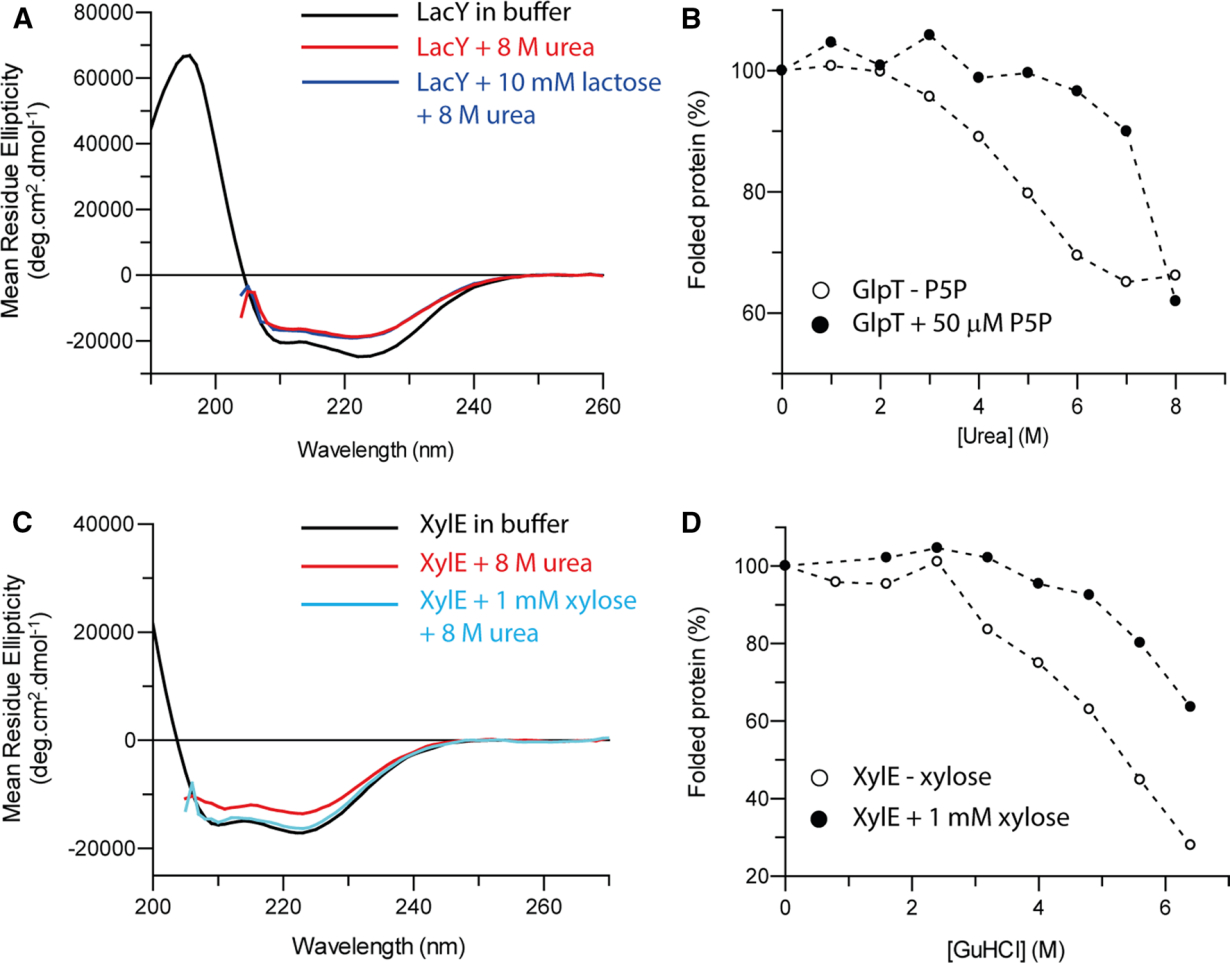
XylE and GlpT are both stabilised by addition of ligand, but LacY-WT is not. **a** LacY-WT loses around a third of its helical structure in 8 M urea (*red line*) compared to its native fold (*black line*). The same unfolding in 8 M urea is observed upon addition of 10 mM lactose (*blue line*). **b** GlpT begins unfolding when the urea concentration is above 2 M, and has a sigmoidal unfolding transition (*white circles*). Addition of 50 µM pyridoxal-5-phosphate during unfolding (*black circles*) stabilises GlpT, causing it to begin unfolding in 6 M urea. XylE is stabilised by addition of xylose in both urea (**c**) and GuHCl (**d**) denaturation. **c** In 8 M urea, XylE loses around 20% of its secondary structure (*red line*) compared to the native fold (*black line*). With the addition of 1 mM xylose, it no longer unfolds (*blue line*). **d** XylE unfolds when the GuHCl concentration is above 2.5 M. Addition of 1 mM xylose during GuHCl denaturation stabilises XylE, and it unfolds above 5 M GuHCl (*black circles*). XylE with xylose still has around 60% of its helical structure remaining in 6 M GuHCl, compared to around 30% without xylose. **b**, **d** are both shown as % folded protein, with 100% being the CD signal at 222 nm in the native fold

CD can also be used to measure the stability of proteins in the presence of ligand, provided that the ligand itself does not have a strong CD signal in the UV region. The transporters LacY, GlpT and XylE were incubated in their relevant ligands prior to chemical denaturation [GalP data are reproduced from a previous study (Findlay et al. 2010)]. When 10 mM lactose (*k*_D_ ~1 mM, Guan and Kaback 2004) is added to LacY-WT during unfolding (Fig. 5a), it did not stabilise against the unfolding induced by 8 M urea. The same experiment repeated with XylE and GlpT however showed the opposite; both were stabilised by addition of their ligands (xylose and pyridoxal-5-phosphate (P5P), respectively). Addition of 50 µM P5P (Santoro et al. 2011) to GlpT during unfolding in urea caused GlpT to begin unfolding when the urea concentration was above 6 M (Fig. 5b), a significant stabilisation. XylE incubated in 8 M urea in the presence of 1 mM xylose (*k*_D_ ~0.35 mM, Sun et al. 2012) did not lose any helical structure, exhibiting the same unfolding behaviour as the urea-resistant MFS transporters PepT_So_ and C154G (Fig. 5c). The addition of 1 mM xylose also stabilised XylE during GuHCl denaturation; XylE with xylose unfolded when the GuHCl concentration was above 5 M, as opposed to 2.5 M without xylose (Fig. 5d; Table 1). Around 60% of the helical structure remained in 6 M GuHCl, double the amount remaining without xylose.

## Discussion

The six MFS transporters studied here fall into two broad categories, the ‘urea-sensitive’ including LacY-WT, GalP and GlpT, and ‘urea-resistant’, including XylE, LacY-C154G and PepT_So_. LacY-C154G and PepT_So_ do not unfold in urea at all, and also do not significantly unfold at low concentrations of the harsher denaturant GuHCl. 4 M GuHCl is required to unfold LacY-C154G and PepT_So_, whereas the other MFS transporters unfold at lower concentrations, around 2 M GuHCl. These results demonstrate that while MFS transporters share a common fold, they do not share the same chemical stability.

One potential mechanism by which the urea-resistant transporters are more stable is decreased flexibility in vitro. The increased thermal stability of LacY-C154G has been ascribed to tighter helical packing induced by the C154G mutation (Ermolova et al. 2005). This tighter packing prevents conformational switching, inactivating transport (Smirnova and Kaback 2003). PepT_So_ and XylE have extra helices between the N and C domains, which are not present in the other transporters in this study (see Fig. 1). The extra helices in PepT_So_, HA and HB, are thought to have a role in maintaining structural rigidity during transport (Zhao et al. 2014). It is possible that the extra helices of PepT_So_ help stabilise the N and C domains, and once HA and HB have unfolded themselves the rest of the protein unfolds, explaining the steep unfolding transition of PepT_So_ above 4 M GuHCl. The extra helices of XylE are known as the ICH domain, and are thought to have a role in holding the intracellular side of the transporter closed. This gating stabilises the outward open conformation (Deng et al. 2014; Nomura et al. 2015; Oka et al. 1990; Sun et al. 2012), which may also decrease the flexibility of XylE and increase the chemical stability. Thus PepT_So_, and to a lesser extent XylE, may be more stable to denaturants as a result of decreased flexibility. The ICH domain of XylE is solvent exposed and therefore readily accessible to denaturant in contrast to the transmembrane HA and HB helices of PepT_so_, perhaps explaining why the stabilising effect is less pronounced, with some unfolding of XylE in urea observed at concentrations above 5 M.

A likely consequence of this decrease in flexibility is to reduce access of the interdomain binding pocket to the denaturant. This has previously been shown to be the case for another urea-resistant LacY mutant, LacY-W151Y, where changes in acrylamide quenching demonstrated that the binding cavity of the mutant was much less accessible to the solvent than is the case for LacY-WT (Harris et al. 2014). XylE and PepT_So_ may also be more stable to denaturants than the urea-sensitive transporters due to a decreased access of denaturants to the hydrophilic binding pocket. PepT_So_ was crystallised in an occluded state (Newstead et al. 2011), with the binding pocket largely inaccessible to the aqueous environment, whereas the urea-sensitive transporters LacY-WT and GlpT were both crystallised in inward-open facing states (Guan et al. 2007; Huang et al. 2003), with the binding pocket accessible (Fig. 1). XylE has been crystallised in a range of conformational states, including an occluded-inward open, occluded-outward open, and inward-open state. The correlation between crystal structure conformation and stability suggests that the conformational state the transporter adopts during crystallisation relates to the conformational state of the protein in vitro. If XylE and PepT_So_ are predominantly in the occluded state in vitro, and thus less accessible to denaturants, that could confer the observed increased stability. In contrast LacY-WT and GlpT were crystallised in an inward-open facing state. Maintaining this conformation in vitro would allow access of denaturants and consequently unfolding. The stabilisation of GlpT by its ligand P5P (Fig. 5b) can be explained as a result of P5P binding either directly blocking part of the binding pocket, or causing a larger conformational switch from the inward-open to an occluded state, in either case preventing access of denaturants. LacY-C154G was crystallised in an inward-open state (Abramson et al. 2003), but a large number of subsequent studies have indicated that this conformation is a crystallographic artefact, and that LacY-C154G is in fact locked in a periplasmic-open state (Majumdar et al. 2007; Nie et al. 2008; Smirnova et al. 2007). As discussed above, the stability of LacY-C154G is well-known to be due the C154G mutation causing tighter helical packing of helices I and V, restricting the flexibility of the transporter (Ermolova et al. 2005), not due to accessibility.

Our results have shown that detergent solubilised MFS transporters have different stabilities to denaturants in vitro, despite their similar structures. Taken together, differences in chemical stabilities of the MFS transporters in this study are correlated with the different conformational states that these proteins occupy as part of the transport cycle, and changes in the ability to move between them. They also relate to the states they were crystallised in; PepT_So_ solved in an occluded state is slightly more stable than XylE, which was solved in a variety of states. Both PepT_So_ and XylE are more stable than LacY and GlpT, which were both solved in inward-open states, suggesting that these detergent solubilised transporters are in a similar conformation in vitro to that crystallised. CD and SRCD have enabled us to compare between different proteins, using the clear reduction in helical structure induced by denaturants to measure unfolding, rather than a protein-specific labelling or fluorescence method, which can be more difficult to interpret and compare between proteins.

## Electronic supplementary material

Below is the link to the electronic supplementary material.

Supplementary material 1 (DOCX 472 kb)

## References

[CR1] Abdul-Gader A, Miles AJ, Wallace BA (2011). A reference dataset for the analyses of membrane protein secondary structures and transmembrane residues using circular dichroism spectroscopy. Bioinformatics.

[CR2] Abramson J, Smirnova I, Kasho V, Verner G, Kaback HR, Iwata S (2003). Structure and mechanism of the lactose permease of *Escherichia coli*. Science.

[CR3] Burck J, Wadhwani P, Fanghanel S, Ulrich AS (2016). Oriented circular dichroism: a method to characterize membrane-active peptides in oriented lipid bilayers. Acc Chem Res.

[CR4] Deng D, Xu C, Sun P, Wu J, Yan C, Hu M, Yan N (2014). Crystal structure of the human glucose transporter GLUT1. Nature.

[CR5] Deng D (2015). Molecular basis of ligand recognition and transport by glucose transporters. Nature.

[CR6] Doki S (2013). Structural basis for dynamic mechanism of proton-coupled symport by the peptide transporter POT. Proc Natl Acad Sci USA.

[CR7] Ermolova NV, Smirnova IN, Kasho VN, Kaback HR (2005). Interhelical packing modulates conformational flexibility in the lactose permease of *Escherichia coli*. Biochemistry.

[CR8] Findlay HE, Rutherford NG, Henderson PJ, Booth PJ (2010). Unfolding free energy of a two-domain transmembrane sugar transport protein. Proc Natl Acad Sci USA.

[CR9] Guan L, Kaback HR (2004). Binding affinity of lactose permease is not altered by the H+ electrochemical gradient. Proc Natl Acad Sci USA.

[CR10] Guan L, Mirza O, Verner G, Iwata S, Kaback HR (2007). Structural determination of wild-type lactose permease. Proc Natl Acad Sci USA.

[CR11] Harris NJ, Findlay HE, Simms J, Liu X, Booth PJ (2014). Relative domain folding and stability of a membrane transport protein. J Mol Biol.

[CR12] Huang Y, Lemieux MJ, Song J, Auer M, Wang DN (2003). Structure and mechanism of the glycerol-3-phosphate transporter from *Escherichia coli*. Science.

[CR13] Lees JG, Smith BR, Wien F, Miles AJ, Wallace BA (2004). CDtool-an integrated software package for circular dichroism spectroscopic data processing, analysis, and archiving. Anal Biochem.

[CR14] Majumdar DS, Smirnova I, Kasho V, Nir E, Kong X, Weiss S, Kaback HR (2007). Single-molecule FRET reveals sugar-induced conformational dynamics in LacY. Proc Natl Acad Sci USA.

[CR15] Newstead S (2011). Crystal structure of a prokaryotic homologue of the mammalian oligopeptide-proton symporters, PepT1 and PepT2. EMBO J.

[CR16] Nie Y, Sabetfard FE, Kaback HR (2008). The Cys154—>Gly mutation in LacY causes constitutive opening of the hydrophilic periplasmic pathway. J Mol Biol.

[CR17] Nomura N (2015). Structure and mechanism of the mammalian fructose transporter GLUT5. Nature.

[CR18] Oka Y (1990). C-terminal truncated glucose transporter is locked into an inward-facing form without transport activity. Nature.

[CR19] Santoro A, Cappello AR, Madeo M, Martello E, Iacopetta D, Dolce V (2011). Interaction of fosfomycin with the glycerol 3-phosphate transporter of *Escherichia coli*. Biochim Biophys Acta.

[CR20] Smirnova IN, Kaback HR (2003). A mutation in the lactose permease of *Escherichia coli* that decreases conformational flexibility and increases protein stability. Biochemistry.

[CR21] Smirnova I, Kasho V, Choe JY, Altenbach C, Hubbell WL, Kaback HR (2007). Sugar binding induces an outward facing conformation of LacY. Proc Natl Acad Sci USA.

[CR22] Solcan N, Kwok J, Fowler PW, Cameron AD, Drew D, Iwata S, Newstead S (2012). Alternating access mechanism in the POT family of oligopeptide transporters. The EMBO journal.

[CR23] Sun L, Zeng X, Yan C, Sun X, Gong X, Rao Y, Yan N (2012). Crystal structure of a bacterial homologue of glucose transporters GLUT1-4. Nature.

[CR24] Ulmschneider MB, Ulmschneider JP, Schiller N, Wallace BA, von Heijne G, White SH (2014). Spontaneous transmembrane helix insertion thermodynamically mimics translocon-guided insertion. Nature Commun.

[CR25] Whitmore L, Wallace BA (2004). DICHROWEB, an online server for protein secondary structure analyses from circular dichroism spectroscopic data. Nucleic Acids Res.

[CR26] Wisedchaisri G, Park MS, Iadanza MG, Zheng H, Gonen T (2014). Proton-coupled sugar transport in the prototypical major facilitator superfamily protein XylE. Nature Commun.

[CR27] Yan N (2015). Structural biology of the major facilitator superfamily transporters. Ann Rev Biophy.

[CR28] Zhao Y, Mao G, Liu M, Zhang L, Wang X, Zhang XC (2014). Crystal structure of the *E. coli* peptide transporter YbgH. Structure.

