# Supplementary material for: Comparative stability of Major Facilitator Superfamily transport proteins
Source: Eur Biophys J. 2017 Jan 23;46(7):655–63. doi: 10.1007/s00249-017-1197-7 (PMC5599477; doi:10.1007/s00249-017-1197-7)
Supplement: Supplementary file 1 — Supplementary material 1 (DOCX 472 kb) [file 249_2017_1197_MOESM1_ESM.docx]

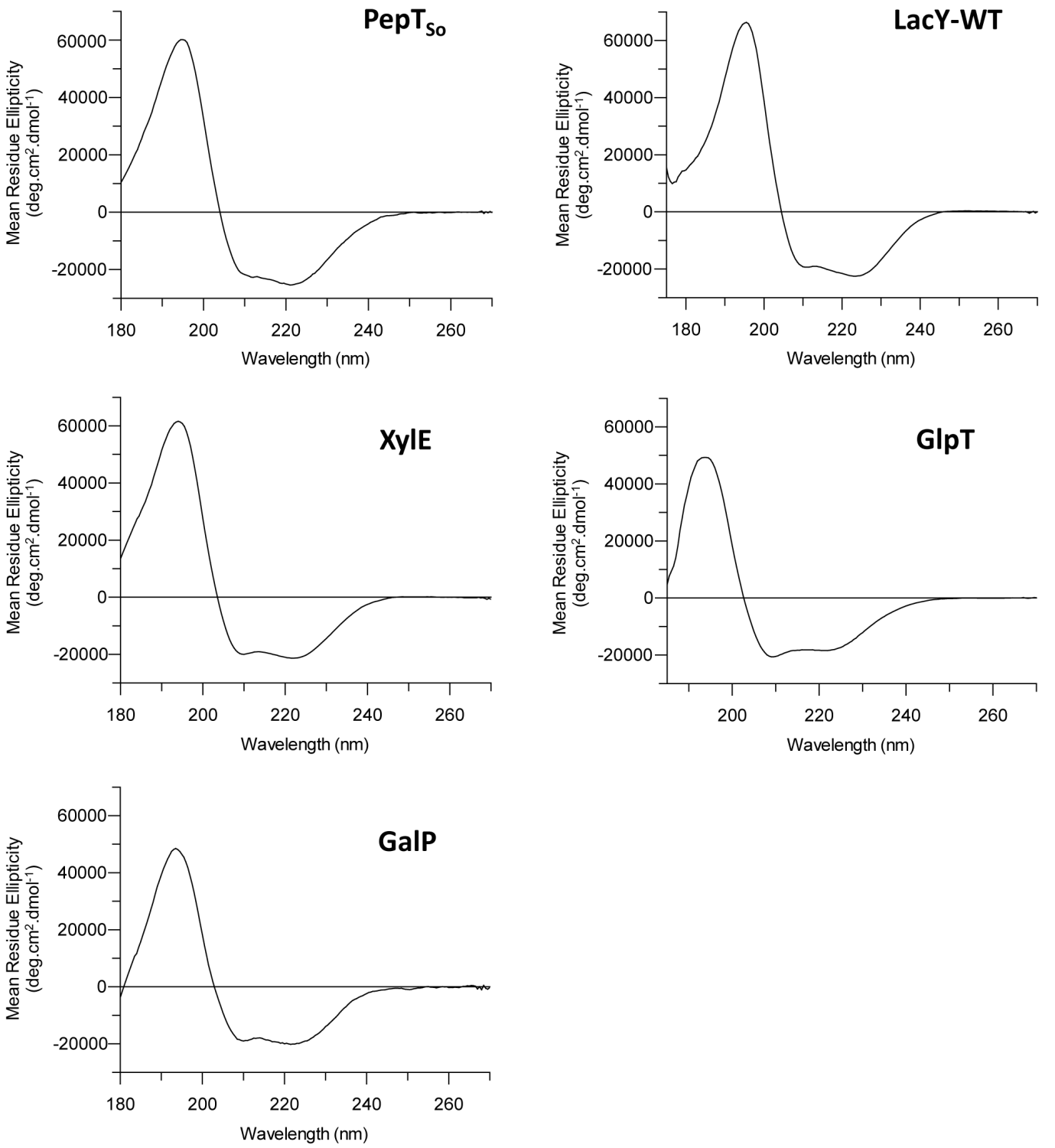


**Fig. S1** Secondary structure of MFS transporters. The MFS transporters have very similar secondary structures, each being highly α-helical. PepT_So_ is 85 % α-helical (measured in a 0.01 mm quartz cell at 3.2 mg.ml^-1^), LacY-WT is 86 % α-helical (measured in a 13 µM CaF_2_ cell at 3.4 mg.ml^-1^), XylE is 92 % α-helical (measured in a 0.05 mm quartz cell at 0.85 mg.ml^-1^), GlpT is 81 % α-helical (measured in a 0.2 mm quartz cell at 0.45 mg.ml^-1^) and GalP is 74 % α-helical (data from ([Findlay et al. 2010](#_ENREF_1)). The spectra are all slightly different shapes, particularly in the ratio between the 208 nm and 222 nm bands. This could reflect differences in the amount of beta turns and unordered structure between the transporters


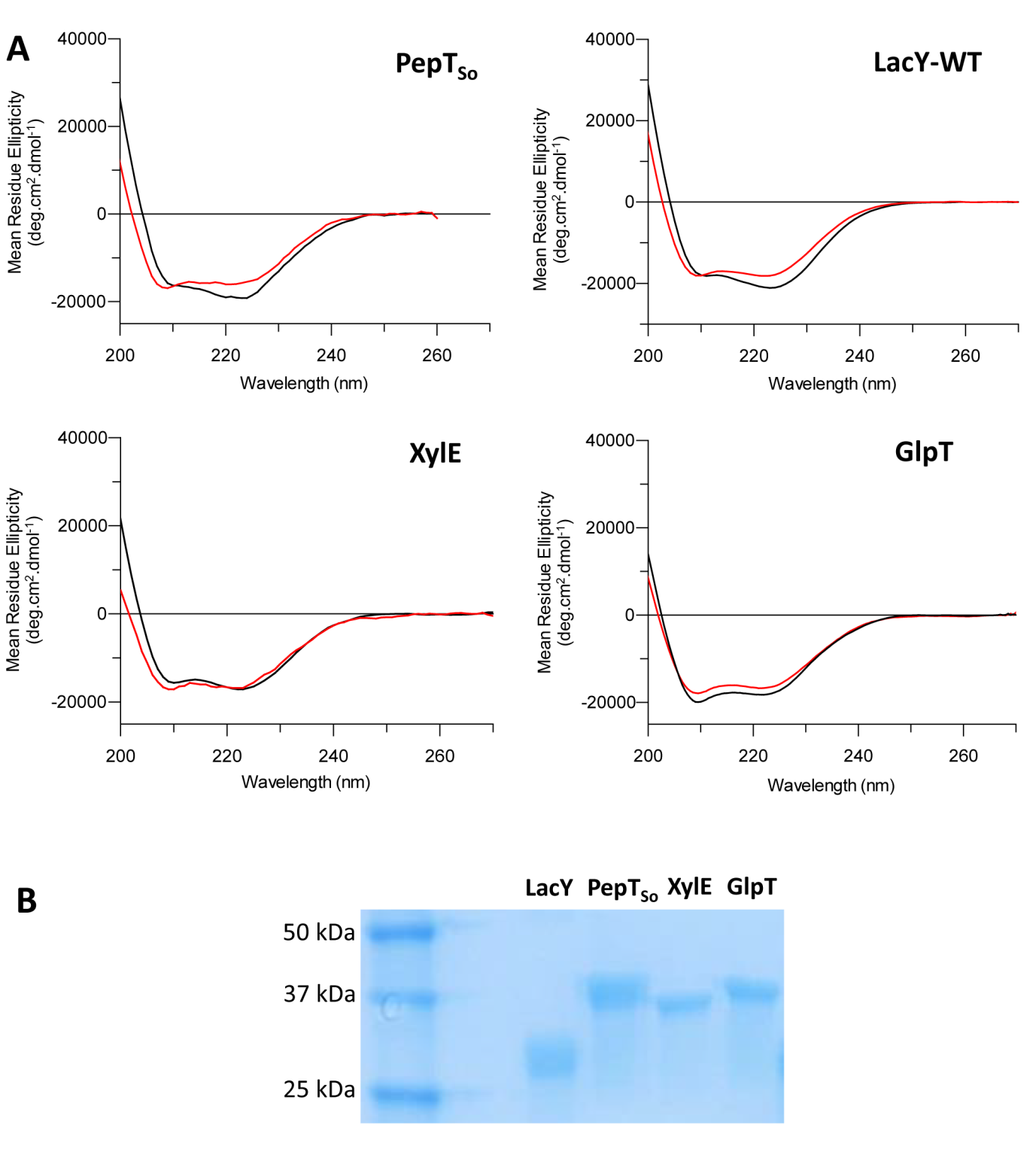


**Fig. S2** MFS transporters do not unfold in SDS. **a** CD spectra of LacY, GlpT, XylE and PepT_So_ incubated in 1 % SDS (apart from XylE, 2 % SDS). They lose little to no secondary structure in SDS (red line) compared to their native structure (black line). The small difference in the shape of the CD spectra likely reflect a slight conformational change in SDS, rather than unfolding. GalP incubated in SDS has been published previously ([Findlay et al. 2010](#_ENREF_1)). **b** When MFS transporters are analysed by SDS-PAGE, they run below their expected molecular weight, an indication that they are still folded. The actual molecular weights of each transporter are as follows: LacY is 48 kDa, PepT_So_ is 60 kDa, XylE is 55 kDa and GlpT is 54 kDa

**References**

Findlay HE, Rutherford NG, Henderson PJ, Booth PJ (2010) Unfolding free energy of a two-domain transmembrane sugar transport protein Proceedings of the National Academy of Sciences of the United States of America 107:18451-18456 doi:10.1073/pnas.1005729107
